# Enhancing Skin Cancer Diagnosis Through Fine-Tuning of Pretrained Models: A Two-Phase Transfer Learning Approach

**DOI:** 10.1155/ijbc/4362941

**Published:** 2025-02-17

**Authors:** Entesar Hamed I. Eliwa

**Affiliations:** Department of Mathematics and Statistics, College of Science, King Faisal University, Al-Ahsa, Saudi Arabia

**Keywords:** fine-tuning, pretrained models, skin cancer classification, transfer learning

## Abstract

Skin cancer is among the most prevalent types of cancer worldwide, and early detection is crucial for improving treatment outcomes and patient survival rates. Traditional diagnostic methods, often reliant on visual examination and manual evaluation, can be subjective and time-consuming, leading to variability in accuracy. Recent developments in machine learning, particularly using pretrained models and fine-tuning techniques, offer promising advancements in automating and improving skin cancer classification. This paper explores the application of a two-phase model using the HAM10000 dataset, which comprises a wide range of skin lesion images. The first phase employs transfer learning with frozen layers, followed by fine-tuning all layers in the second phase to adapt the models more specifically to the dataset. I evaluate nine pretrained models, including VGG16, VGG19, InceptionV3, Xception (extreme inception), and DenseNet121, assessing their performance based on accuracy, precision, recall, and *F*1 score metrics. The VGG16 model, after fine-tuning, achieved the highest test set accuracy of 99.3%, highlighting its potential for highly accurate skin cancer classification. This study provides important insights for clinicians and researchers, demonstrating the efficacy of advanced machine learning models in enhancing diagnostic accuracy and supporting clinical decision-making in dermatology.

## 1. Introduction

The skin, being the body's largest organ, serves as the primary defense against external elements, protecting against pathogens and harmful ultraviolet (UV) radiation. However, as the outermost layer, it is vulnerable to various conditions, including skin cancer, which is among the most prevalent cancers globally. “Skin cancer” encompasses a range of diseases characterized by uncontrolled growth of abnormal skin cells, leading to tumors. UV radiation exposure is a major risk factor for these cancers [1].

The three main types of skin cancer are melanoma (MEL), basal cell carcinoma (BCC), and squamous cell carcinoma. Actinic keratoses (AKs) and actinic keratosis intraepithelial carcinoma (AKIEC) are precancerous and early malignant conditions caused by prolonged UV exposure. BCC is the most common type, usually presenting as pearly or waxy bumps. MEL is highly aggressive, originating from melanocytes, and capable of metastasizing if untreated. Noncancerous growths like benign keratosis-like lesions (BKL), dermatofibromas (DFs), melanocytic nevi (NVs), and vascular lesions (VASC) need careful evaluation to exclude malignancy or address cosmetic concerns [2].

Differentiating between these conditions is crucial for determining treatment strategies and prognosis. Early detection and intervention for AK and AKIEC can prevent progression to invasive carcinoma, while timely treatment of BCC and MEL can reduce metastasis risk and improve patient outcomes. Proper evaluation of benign lesions is essential to avoid unnecessary procedures and address patient concerns effectively [3].

Early detection of skin cancer significantly improves treatment success and survival rates. Traditionally, dermatologists diagnose skin conditions through visual examinations, often enhanced by dermoscopy, a noninvasive technique that magnifies the skin's surface. However, these methods rely heavily on the clinician's expertise, which can sometimes lead to subjective evaluations [4].

The diagnostic process involves several steps. Initially, the dermatologist performs a visual inspection to identify abnormal growths. This is followed by dermoscopy, which uses a magnifying lens to examine lesion patterns closely. The final step often involves a biopsy to obtain a tissue sample for further analysis [5–7].

Before dermoscopic imaging became widespread, even skilled dermatologists had a success rate of around 60% in diagnosing skin cancer. The advent of dermoscopic imaging has improved these rates to between 75% and 84%. Accurate diagnosis still depends on the clinician's expertise, and manual diagnosis can be challenging and stressful for patients. In situations where professional or diagnostic expertise is lacking, computer-aided detection systems can assist by analyzing dermoscopic data [8].

Machine learning and deep learning technologies have greatly enhanced skin cancer detection by enabling automatic, accurate, and efficient analysis of skin lesions and images. These technologies support early detection, maintain consistent performance, and facilitate comprehensive data analysis, making them valuable for widespread use. They help reduce unnecessary diagnoses and ensure timely identification of potential skin cancer cases [9].

### 1.1. Problem Statement

Skin cancer remains a leading cause of cancer-related deaths globally, and early and accurate diagnosis is crucial for effective treatment and improved patient outcomes. Traditional skin cancer classification methods, relying on visual inspection and manual assessment, are subject to variability in accuracy and can be time-consuming. Recent advancements in machine learning, particularly through the use of pretrained models and fine-tuning techniques, have shown promise in enhancing the accuracy and efficiency of skin cancer classification. However, there is a need to systematically evaluate these advancements by comparing classification times and performance metrics. The existing literature lacks comprehensive analyses on how fine-tuning pretrained models affects both classification accuracy and processing efficiency in the context of a skin cancer diagnosis.

### 1.2. Research Motivation

The motivation for this research stems from the critical need to improve skin cancer diagnosis methods to enhance patient outcomes and streamline clinical workflows. By leveraging advanced pretrained models and employing fine-tuning techniques, this study is aimed at addressing two key aspects.

### 1.3. Accuracy Improvement

Pretrained models, such as those based on deep learning architectures, have demonstrated superior performance in various image classification tasks. Fine-tuning these models on specialized skin cancer datasets has the potential to significantly improve classification accuracy and diagnostic precision.

This research seeks to bridge the gap between theoretical advancements in machine learning and practical applications in skin cancer diagnosis, ultimately contributing to more efficient and accurate diagnostic processes. By evaluating both the performance and time efficiency of fine-tuned pretrained models, this study is aimed at providing valuable insights for healthcare professionals and researchers in the field.

### 1.4. Contributions

I can summarize the contributions of this paper as follows:

• Two-phase transfer learning model: proposes an innovative two-phase model utilizing transfer learning to enhance skin cancer classification accuracy and performance.
• Evaluation of advanced models: conducts a thorough evaluation of nine state-of-the-art pretrained models to determine their effectiveness in skin cancer detection.
• Superior performance: demonstrates that the proposed model outperforms existing methods reported in the literature, achieving higher accuracy and improved classification results.
• In-depth performance metrics: provides a comprehensive analysis of various performance metrics, including precision, recall, and *F*1 score, in addition to accuracy, to offer a more holistic assessment of model effectiveness.

## 2. Background

I provide an overview of each pretrained model architecture utilized in this study, emphasizing their distinctive features and their impact on advancing image classification techniques.

### 2.1. VGG16 and VGG19

Developed by the Visual Geometry Group (VGG) at the University of Oxford, VGG16 and VGG19 are renowned for their simplicity and uniform architecture. Both models utilize 3 × 3 convolutional layers stacked on top of each other, with VGG16 comprising 16 weight layers and VGG19 containing 19. This design enables deep feature extraction while maintaining a consistent structure throughout the network. Despite their depth, VGG models are computationally intensive, which can be a consideration for practical applications [10].

### 2.2. InceptionV3

Introduced by Google, InceptionV3 is part of the Inception series of architectures designed to efficiently capture spatial hierarchies in images. It employs a combination of convolutions with multiple filter sizes within the same module, allowing the network to process information at different scales simultaneously. This approach enhances the model's ability to recognize complex patterns while optimizing computational resources [11].

### 2.3. Xception (Extreme Inception)

Xception is an extension of the Inception architecture that replaces standard inception modules with depthwise separable convolutions. This modification leads to a more efficient model with fewer parameters, improving performance on various image classification tasks. Xception's architecture allows for capturing cross-channel correlations and spatial correlations independently, enhancing its feature extraction capabilities [12].

### 2.4. ResNet152V2

ResNet152V2 is a deep residual network that addresses the vanishing gradient problem commonly encountered in very deep networks. It introduces residual connections, or shortcuts, that allow gradients to flow more easily through the network during training. This design enables the construction of significantly deeper models, with ResNet152V2 containing 152 layers, leading to improved accuracy in image classification tasks [12].

### 2.5. DenseNet121 and DenseNet201

DenseNet architectures, including DenseNet121 and DenseNet201, connect each layer to every other layer in a feed-forward fashion. This dense connectivity pattern ensures maximum information flow between layers, leading to improved feature reuse and gradient propagation. As a result, DenseNets often require fewer parameters and can be more efficient than traditional convolutional networks of similar depth [13].

### 2.6. MobileNet and MobileNetV2

Designed for mobile and embedded vision applications, MobileNet and its successor MobileNetV2 prioritize model efficiency and low computational cost. They utilize depthwise separable convolutions to reduce the number of parameters and computations, making them suitable for deployment on devices with limited resources. Despite their lightweight nature, MobileNet architectures maintain competitive accuracy in image classification tasks [10].

These architectures have been widely adopted in various image classification tasks, including medical imaging, due to their unique strengths and adaptability to transfer learning. Understanding their foundational principles provides insight into their selection and application in this study.

## 3. Related Work

Ayesha et al. [10] presented the multimodel fusion for skin cancer detection (MMF-SCD), a deep learning model designed to improve the classification of skin cancer types using the ISIC dataset, which consists of over 10,000 dermoscopic images. Their methodology included data augmentation and image preprocessing, which involved resizing the images to 224 × 224 pixels and rescaling pixel values. They also performed feature extraction using three pretrained convolutional neural networks (CNNs): VGG16, VGG19, and ResNet-50. The model achieved a high accuracy rate of 97.6%, with precision at 97%, recall at 96%, and an *F*1 score of 96%. These results indicated that the MMF-SCD model significantly outperformed previous methodologies, which had accuracy rates ranging from 82% to 96%. The research highlighted the potential of deep learning techniques in enhancing diagnostic accuracy for skin cancer detection, providing a practical solution to classification challenges in dermatology. The study also suggested that this approach could be extended to other medical imaging applications, such as lung cancer and brain tumor detection, indicating a broader impact on healthcare diagnostics.

Venugopal et al. [14] introduced a deep neural network (DNN) model that utilized a modified EfficientNet for skin cancer detection in dermoscopic images, aiming to enhance early diagnosis and reduce misdiagnosis. They employed a comprehensive knowledge base constructed from 58,032 refined dermoscopic images sourced from the ISIC 2020, ISIC 2019, and ISIC 2018 datasets. Their methodology involved transfer learning and fine-tuning techniques to optimize model training with limited data, alongside data augmentation strategies to boost performance. The DNN model classified skin lesions into seven categories, which were then aggregated into binary classes (malignant and benign) for skin cancer detection, highlighting the potential of AI in assisting dermatologists with computer-aided diagnosis (CAD) and paving the way for improved skin cancer detection methodologies. The study's results on the HAM10000 dataset showed that the modified EfficientNetV2-M model achieved an accuracy of 95.95% for the binary classification of skin lesions into malignant and benign categories. Additionally, the model demonstrated a high AUC (area under the curve) score of 0.98, reflecting its strong ability to distinguish between the two classes. In multiclass classification, the model attained an accuracy of 94.80%, showcasing its effectiveness in identifying various skin lesion types.

Alenezi, Armghan, and Polat [15] introduced a wavelet transform–based deep residual neural network (WT-DRNNet) for skin lesion classification, focusing on enhancing diagnostic accuracy. They employed a methodology divided into three phases: preprocessing, feature extraction, and classification. In the preprocessing phase, they applied wavelet transformation, pooling, and normalization to improve image details and remove unwanted artifacts. Feature extraction utilized a pretrained ResNet101 architecture to derive deep features, which were then classified using an extreme learning machine (ELM) with a ReLU (rectified linear unit) activation function. The model was tested on the HAM10000 dataset, which contained 10,015 images across seven skin lesion classes. The best performance metrics achieved included 95.73% accuracy, 98.8% specificity, 95.84% precision, and 93.44% *F*1 score. This approach highlighted the potential of integrating advanced deep learning techniques with image processing methods to enhance diagnostic capabilities in dermatology.

Jasil and Ulagamuthalvi [16] presented a hybrid CNN architecture for skin lesion classification using the HAM10000 dataset, which comprised 10,015 dermatoscopic images of various skin lesions. They employed a methodology that combined DenseNet and residual networks to integrate contextual data and enhance classification accuracy. The dataset was preprocessed, resized to 224 × 224 pixels, and augmented with techniques such as flipping, rotation, and adding Gaussian noise to address the class imbalance and prevent overfitting. After training the model for 100 epochs, it achieved an impressive accuracy of 95% on the HAM10000 dataset. The performance evaluation metrics included accuracy, sensitivity, specificity, and precision, with the model demonstrating strong classification capabilities across seven skin lesion classes: AK, nevus, BCC, BKL, dermato broma, MEL, and VASC.

Bozkurt [17] focused on skin lesion classification using a pretrained deep learning approach and effective data augmentation techniques. They utilized the HAM10000 dataset, which comprised 10,015 dermatoscopic images of pigmented lesions across seven classes. The methodology involved applying affine transformation techniques for data augmentation, increasing the dataset size to 39,787 images. A hybrid network model, Inception-ResNet-v2, was employed for classification. The best results achieved with the augmented dataset using the Inception-ResNet-v2 model yielded an accuracy of 95.09%. In contrast, the model achieved an accuracy of 83.59% with the original dataset.

Tahir et al. [5] presented the DSCC_Net model, a deep learning–based approach for diagnosing skin cancer using dermoscopic images. They evaluated the model on three publicly available benchmark datasets: ISIC 2020, HAM10000, and DermIS. The methodology involved a CNN architecture with five convolutional blocks and utilized the SMOTE Tomek technique to address the class imbalance in the dataset. For the HAM10000 dataset, specifically the DSCC_Net model, notable results were achieved, with metrics including an accuracy of 94.17%, precision of 94.28%, recall of 93.76%, *F*1 score of 93.93%, and an AUC of 99.43%. These metrics highlighted the model's high performance in accurately classifying various skin cancer types, such as MEL, BCC, squamous cell carcinoma, and NVs. The study emphasized the importance of automated systems in enhancing diagnostic accuracy and efficiency in healthcare settings.

Aydin [18] focused on skin cancer detection using histogram-based local descriptors. They employed two datasets: Dataset 1 from Kaggle, which classified skin cancer as benign or malignant, and the HAM10000 dataset, containing 10,015 images categorized into seven classes. The methodology involved feature extraction using local descriptors such as local binary pattern (LBP), local directional number pattern (LDN), pyramid of histogram of oriented gradients (PHOGs), local directional pattern (LDiP), and monogenic binary coding (MBC), followed by classification with support vector machines (SVMs) and XGBoost. For the HAM10000 dataset, the best results were achieved using the colored MBC feature with the XGBoost classifier, attaining an accuracy of 96.50% and an *F*1 score of 96.48%. The study highlighted the effectiveness of colored features in enhancing skin cancer classification, suggesting that these methods could be beneficial in clinical settings for early detection and diagnosis.

Hosseinzadeh et al. [19] employed a comprehensive approach by integrating various machine learning methodologies, utilizing datasets like ISBI2016, ISBI2017, and HAM10000, which are well-regarded in dermatology for skin lesion classification and provide a reliable basis for model evaluation. The researchers implemented several key steps in their methodology, including preprocessing for image quality enhancement, feature extraction to identify significant image characteristics, and feature selection using the Lasso method to improve model performance by focusing on the most relevant features. The study then combined multiple algorithms—such as XGBoost, multilayer perceptron (MLP), SVM, and random forest—using a stacking approach to leverage the strengths of each. The model achieved notable results, with an accuracy of 87.72% and a sensitivity of 92.15%.

Musthafa et al. [4] focused on enhancing skin cancer diagnosis through an optimized CNN architecture. They utilized the HAM10000 dataset, which contains over 10,000 dermatoscopic images and includes seven distinct categories of skin cancer: AK, intraepithelial carcinoma/Bowen's disease, BCC, BKL, DF, MEL, and NVs. This dataset was crucial for developing machine learning models for diagnosing pigmented skin lesions. The methodology involved several steps, starting with data preprocessing to enhance image quality and standardize inputs for the CNN. The model was trained using techniques like dropout and early stopping to prevent overfitting, ensuring generalizability to unseen data. Extensive data augmentation, including rotations, zooming, and flipping, was applied to further enhance the model's ability to generalize. The study reported the following performance metrics for the optimized CNN model: an accuracy of 97.78%, precision of 97.9%, recall of 97.9%, and an *F*1 score of 97.8%.

Nagadevi, Suman, and Lakshmi [20] presented an enhanced model for skin lesion detection and classification using a hybrid convolution–based ensemble learning approach combined with advanced segmentation techniques. They focused on improving early detection and accurate classification of skin lesions, particularly MEL, which poses a significant health concern. The study utilized two key datasets: the HAM10000 dataset and the PH2 dataset, which includes various features related to skin lesions, such as blue-whitish veil, dots/globules, and clinical diagnoses. The methodology involved collecting dermoscopic images from these datasets, followed by segmenting the images using a dilated mask RCNN (region-based convolutional neural network) with an attention mechanism to identify abnormal regions. The segmented images were then classified using an adaptive hybrid convolution–based ensemble learning (AHC-EL) model, which incorporated techniques like residual attention network (RAN), MobileNet, and Inception. A hybrid optimization algorithm named fitness-aided battle royale and red deer algorithm (FBR-RDA) was employed to enhance classification performance by tuning parameters such as optimizer, activation function, batch size, and epochs. The model achieved an accuracy of 94.19% on the HAM10000 dataset and 97.33% on the PH2 dataset, demonstrating significant improvements in classification metrics.

Attallah [11] built the Skin-CAD system, which demonstrated remarkable performance across various datasets for skin cancer classification. For the “Skin Cancer: Malignant vs. Benign” dataset, Attallah's system achieved an accuracy of 97.2%, with a specificity of 96.93% and a sensitivity of 97.17%, showcasing its ability to accurately classify malignant and benign cases with minimal false negatives. In the HAM (human-annotated mapping) dataset, Skin-CAD also excelled, attaining the highest accuracy among the evaluated systems, though it exhibited slightly lower sensitivity compared to the previous dataset. In contrast, the ISIC 2018 dataset yielded an accuracy of 85.4%, indicating effective classification but at a lower performance level compared to Skin-CAD. On the other hand, the ISIC 2019 dataset demonstrated outstanding results with an accuracy of 98.80%, reflecting the effectiveness of the methodologies used in classifying skin lesions. Additional noteworthy results include the faster R-CNN on the ISIC 2020 dataset, which achieved an accuracy of 98.5% in distinguishing MEL from benign tumors, and ResNet on the HAM10000 dataset, which recorded the second-highest accuracy of 95.73%, although it was less efficient in specificity and precision compared to Skin-CAD. ResNet-50 showed the lowest accuracy of 88.50% on the HAM10000 dataset, highlighting its relative inefficiency. These outcomes underscore the advancements in CAD for skin cancer, with Attallah's Skin-CAD model emerging as the superior tool across multiple datasets.

Table 1 summarizes the relevant previous studies on skin cancer detection models, detailing their methodologies and best performance metrics.

## 4. Methodology

**Figure 1:**
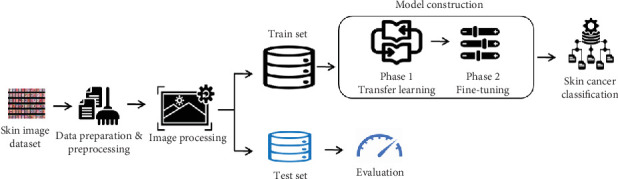
Framework for two-phase skin cancer classification.

This section delineates the methodology adopted in this research to develop and evaluate the two-phase model for classifying skin lesions using the HAM10000 dataset.  Figure 1 summarizes the proposed methodology.

### 4.1. Dataset Description

**Figure 2:**
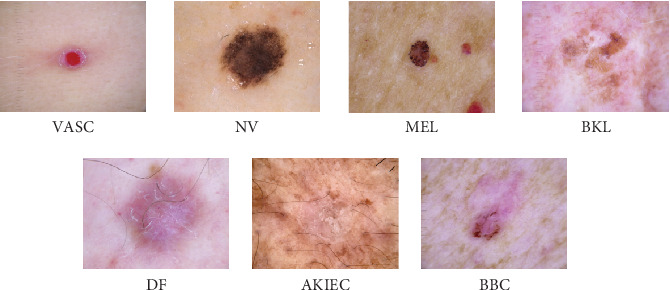
A sample of images from the HAM10000 dataset showing different types of skin lesions.

In this paper, the HAM10000 dataset was utilized to classify skin cancer [21]. This dataset, available through the ISIC archive, contains 10,015 images divided into seven subclasses of skin lesions: 142 images of VASC, 6705 of NV, 1113 of MEL, 1099 of BKL, 115 of DF, 327 of AK (AKIEC), and 514 of BCC. The diversity of classes allows for comprehensive training of classification models, although the class imbalance, particularly the higher number of benign lesions compared to malignant ones, needs to be considered to avoid introducing bias into the model.  Figure 2 displays a variety of images obtained from the HAM10000 dataset.

### 4.2. Data Preparation and Preprocessing

The dataset was compiled by consolidating images from two separate directories into a single, cohesive set. Image IDs were extracted from filenames, and a mapping was created to associate these IDs with their respective file paths. Additionally, human-readable labels for different lesion types were established to facilitate the analysis and classification process. The dataset was then loaded into a structured format and underwent initial exploration to assess its contents and quality. This included viewing samples of the data and identifying any missing or incomplete entries. The data was subsequently processed to ensure consistency and readiness for the next step.

### 4.3. Image Processing

Images in the dataset were standardized to ensure uniformity in their size and format, which is crucial for input into the neural network. This involved resizing the images and converting them into a suitable numerical format that could be utilized by the model.

### 4.4. Model Construction

#### 4.4.1. Phase 1: Transfer Learning With Frozen Layers

• Initial training: The fully connected layers of each pretrained model are replaced with a new set of dense layers appropriate for the classification task. These layers are trained while keeping the convolutional base frozen. The goal is to train the model to learn task-specific features while retaining the generic image features learned from the pretrained models.

#### 4.4.2. Phase 2: Fine-Tuning With Unfrozen Layers

• Unfreezing layers: After the initial training phase, the convolutional base layers of the pretrained models are unfrozen, and the entire model is trained with a lower learning rate. This step allows the model to fine-tune the pretrained weights in conjunction with the newly added dense layers, making the model more attuned to the nuances of the skin cancer dataset.
• Model architecture: The model was structured as a CNN, designed to handle the complexities of image classification tasks, particularly for distinguishing between various types of skin lesions. The architecture was composed of several key layers, each playing a distinct role in the feature extraction and classification process.
• Convolutional layers: The architecture began with a series of convolutional layers, which were responsible for detecting local patterns within the images, such as edges, textures, and more complex features. These layers applied filters to the input images, creating feature maps that emphasized different aspects of the visual data. The filters were learned during the training process, allowing the model to automatically adjust and optimize the features it detects.
• Pooling layers: Following the convolutional layers, pooling layers were used to reduce the spatial dimensions of the feature maps. This downsampling process helped to decrease the computational load and mitigate overfitting by making the model less sensitive to small variations in the input data. The pooling layers selected the most prominent features from each region, preserving the most critical information while discarding less relevant details.
• Fully connected layers: After the feature extraction by the convolutional and pooling layers, the output was flattened and passed through fully connected layers. These layers combined the extracted features to form more abstract representations, ultimately leading to the final classification. The fully connected layers were responsible for making the decisions about which class the input image belonged to, based on the patterns learned during training.
• Dropout regularization: To prevent overfitting, dropout layers were incorporated within the fully connected layers. During training, these layers randomly deactivated a certain percentage of neurons, forcing the network to learn more robust features that were not overly dependent on specific pathways. This regularization technique helped to improve the generalizability of the model.
• Output layer: The final layer of the architecture was a softmax layer, which produced a probability distribution over the possible classes. This layer allowed the model to output a confidence score for each class, facilitating the classification decision. The class with the highest probability was selected as the model's prediction for the given input image.

This architecture, with its combination of convolutional layers for feature extraction and fully connected layers for classification, was well suited to the task of identifying and differentiating between various skin lesions in the dataset. The use of dropout regularization further ensured that the model would perform reliably on new, unseen data.

### 4.5. Training Process

• Optimizer and loss function: The model uses an optimizer like Adam or SGD (stochastic gradient descent) with a learning rate scheduler to adjust the learning rate dynamically during training. The categorical cross-entropy loss function is used due to the multiclass nature of the skin cancer classification problem.
• Early stopping and checkpoints: The training process includes callbacks like early stopping to prevent overfitting and model checkpoints to save the best model weights based on validation performance.

### 4.6. Evaluation Metrics

• Performance metrics: The model's performance is evaluated using metrics such as accuracy, precision, recall, and *F*1 score. These metrics provide a comprehensive view of the model's ability to classify skin cancer images correctly.
• Confusion matrix: A confusion matrix is also plotted to visualize the performance across different classes, giving insights into which classes are being misclassified.

### 4.7. Testing and Validation

• Test set evaluation: After training, the final model is evaluated on a separate test set to ensure its generalizability to unseen data.
• Comparison of models: The performance of different pretrained models is compared to select the best performing model for skin cancer classification.

## 5. Experimental Results

To assess the effectiveness of our proposed method for skin cancer classification, I implemented it across nine widely used pretrained models. These models include VGG16, VGG19, InceptionV3, Xception, DenseNet121, DenseNet201, ResNet152V2, MobileNet, and MobileNetV2. These models were chosen for their architecture's adaptability to transfer learning and their historical success in medical imaging studies [22, 23].

The results for each model are presented in the following sections.

### 5.1. VGG16

**Figure 3:**
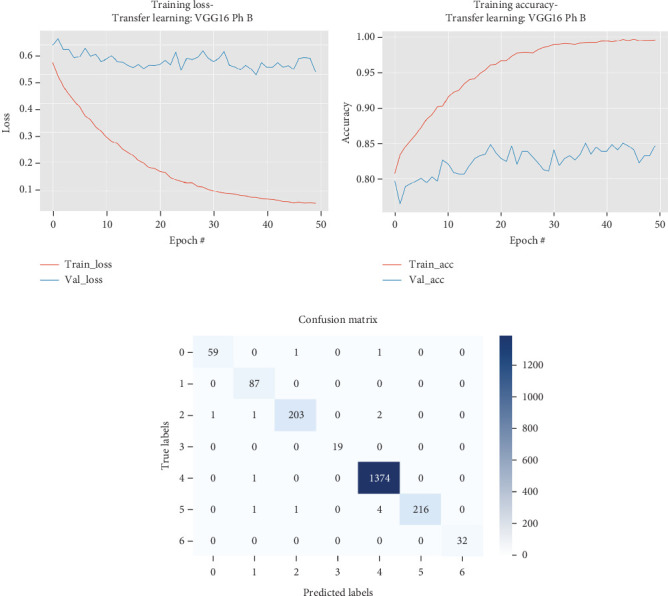
Accuracy and loss plots, along with the confusion matrix for the VGG16 model.

The results for the VGG16 model are presented in Table 2 and illustrated in Figure 3.

As shown in Table 2 and Figure 3, the VGG16 model underwent a two-phase training process, with Phase A involving transfer learning with frozen layers and Phase B involving fine-tuning with unfrozen layers. In Phase A, the model quickly achieved high training accuracy but displayed fluctuating validation accuracy, indicating potential overfitting, as evidenced by the divergence between training and validation loss curves. Despite this, the model reached a maximum validation accuracy of 0.7844 and a test accuracy of 0.8572, with precision, recall, and *F*1 scores all around 0.857, showing balanced performance. In Phase B, fine-tuning significantly improved both training and validation performance. The model achieved a higher maximum validation accuracy of 0.8503 and an impressive test accuracy of 0.9935, with precision, recall, and *F*1 scores close to 0.9935, reflecting near-perfect classification capability. The substantial increase in performance during fine-tuning suggests that unlocking the deeper layers allowed the model to better adapt to the dataset, reducing overfitting and improving generalization, demonstrating the effectiveness of the two-phase strategy for optimizing the model's performance on skin cancer classification.

### 5.2. VGG19

**Figure 4:**
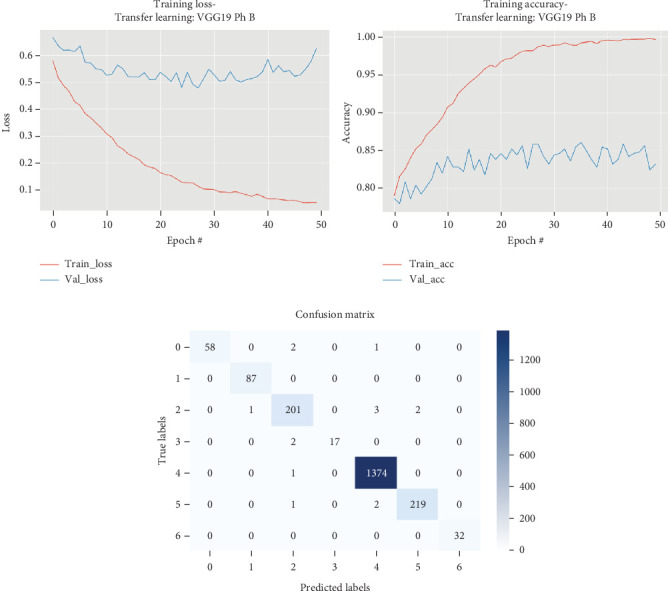
Accuracy and loss plots, along with the confusion matrix for the VGG19 model.

The results for the VGG19 model are presented in Table 3 and illustrated in Figure 4.

Table 3 and Figure 4 illustrate that in Phase A of training the VGG19 model, the maximum validation accuracy achieved was 0.7844, with a training time of 497.24 s and a testing time of 2.3 s, resulting in a test set accuracy of 0.8532, precision of 0.8574, recall of 0.8532, and an *F*1 score of 0.8436. During Phase B, where all layers were fine-tuned, the training time significantly increased to 1309.95 s. However, this phase resulted in a substantial improvement in model performance, achieving a maximum validation accuracy of 0.8603, a test set accuracy of 0.9925, precision of 0.9925, recall of 0.9925, and an *F*1 score of 0.9925, all within a testing time of 2.32 s. The detailed epoch-by-epoch results show progressive improvements in both training and validation loss, with significant gains in accuracy as the training progressed. By Epoch 50, the model consistently maintained high accuracy, demonstrating the effectiveness of fine-tuning all layers in enhancing the model's ability to generalize better on unseen data.

### 5.3. InceptionV3

**Figure 5:**
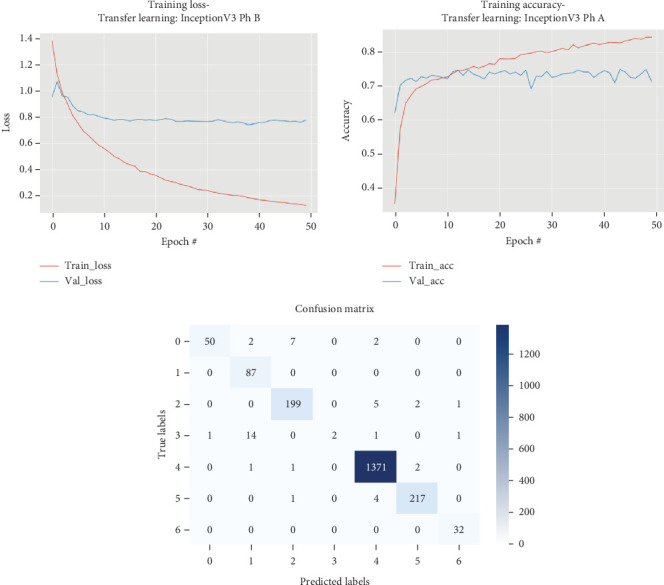
Accuracy and loss plots, along with the confusion matrix for the InceptionV3 model.

The results for the InceptionV3 model are presented in Table 4 and illustrated in Figure 5.

Table 4 and Figure 5 indicate that in Phase B, the InceptionV3 model underwent fine-tuning of all layers, which significantly enhanced its performance over the course of training. Initially, the model began with a high training loss of 1.3825 and moderate accuracy of 0.6231, while the validation loss and accuracy were 0.9547 and 0.6866, respectively. With continued training, there was a marked improvement in both training and validation metrics. By Epoch 10, the model's training loss had decreased to 0.5862 with an accuracy of 0.7958, and the validation loss and accuracy had improved to 0.8049 and 0.7345. As training progressed further, the model consistently demonstrated better performance. By Epoch 25, the training loss was reduced to 0.3000 with an accuracy of 0.9131, and the validation loss and accuracy were 0.7670 and 0.7465, respectively. By the final epoch recorded, Epoch 35, the model achieved a training loss of 0.2072 and an accuracy of 0.9497, with a validation loss of 0.7621 and a validation accuracy of 0.7485. Overall, fine-tuning all layers led to substantial improvements in both training and validation outcomes, underscoring the effectiveness of this approach in enhancing the InceptionV3 model's performance.

### 5.4. Xception

**Figure 6:**
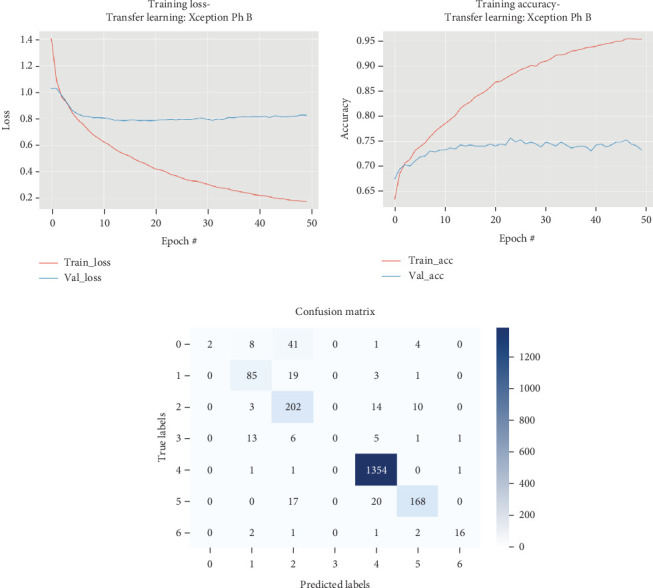
Accuracy and loss plots, along with the confusion matrix for the Xception model.

The results for the Xception model are presented in Table 5 and illustrated in Figure 6.

As presented in Table 5 and Figure 6, during Phase B of the Xception model fine-tuning, all layers were trained to improve performance. This phase achieved a maximum validation accuracy of 75.45% and a test set accuracy of 95.38%, with a precision of 93.87%, recall of 95.38%, and an *F*1 score of 93.83%. The training time for this phase was approximately 1637 s, while testing took around 2.6 s. Throughout the 50-epoch training process, the model's loss decreased progressively, and the accuracy improved, demonstrating effective learning and adaptation. Starting with a training loss of 1.4007 and accuracy of 63.43%, the model consistently improved its validation loss from 1.0282 to a minimum of 0.7833 by Epoch 20. The accuracy also showed steady progress, reaching over 92% by the final epochs, indicating robust model performance on unseen data. The continuous improvement in validation loss and accuracy highlights the model's capacity to generalize well beyond the training set, making it highly effective for the task at hand.

### 5.5. DenseNet121

**Figure 7:**
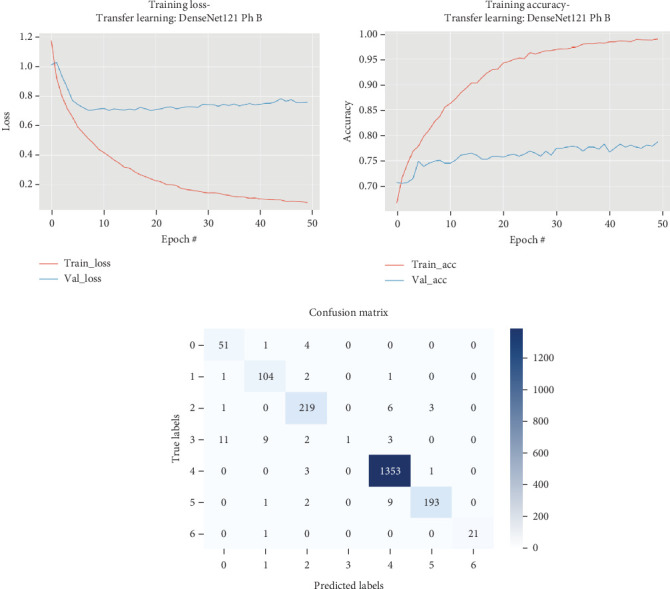
Accuracy and loss plots, along with the confusion matrix for the DenseNet121 model.

The results for the DenseNet121 model are presented in Table 6 and illustrated in Figure 7.

Table 6 and Figure 7 highlight that the DenseNet121 model underwent two phases of training, each demonstrating distinct performance metrics. In Phase A, the model required 317.56 s for training, achieving a maximum validation accuracy of 0.7924. The subsequent testing phase took 4.34 s, with the model attaining a test set accuracy of 0.8582, a precision of 0.8576, a recall of 0.8582, and an *F*1 score of 0.8470. Phase B saw an extended training period of 948.15 s, where the model reached a slightly lower maximum validation accuracy of 0.7864. However, it showed improved results during testing, taking 3.88 s and achieving a test set accuracy of 0.9695. Precision and recall during this phase were 0.9709 and 0.9695, respectively, with an *F*1 score of 0.9642. Throughout 50 epochs, notable improvements in training accuracy and loss were observed, particularly in the initial epochs, where the model consistently improved its validation loss. Despite some fluctuations, the model achieved a final validation loss of 0.70097 by Epoch 20, with a corresponding validation accuracy of 0.7585. However, from Epoch 21 onwards, the validation loss did not improve, suggesting that the model had reached its optimal performance threshold.

### 5.6. DenseNet201

**Figure 8:**
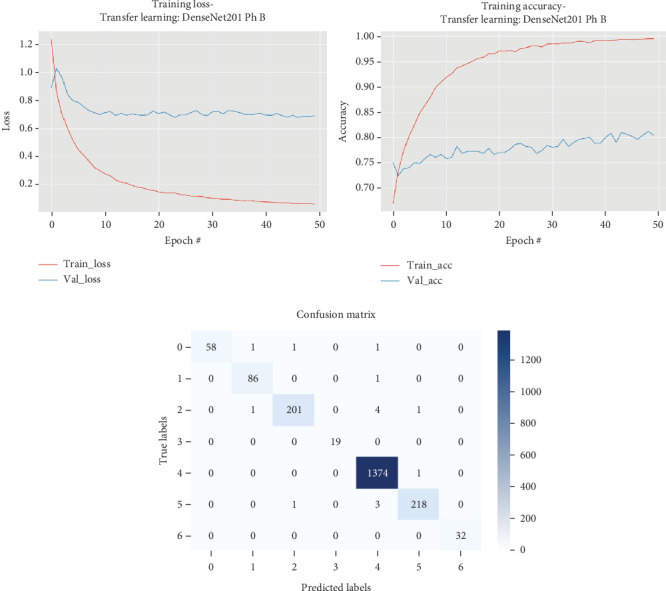
Accuracy and loss plots, along with the confusion matrix for the DenseNet201 model.

The results for the DenseNet201 model are presented in Table 7 and illustrated in Figure 8.

As illustrated in Table 7 and Figure 8, in Phase A, the DenseNet201 model exhibited a training time of approximately 511.59 s, achieving a maximum validation accuracy of 0.8104 and a test set accuracy of 0.8992 with precision, recall, and *F*1 score values of 0.8983, 0.8992, and 0.8944, respectively. During Phase B, the training time significantly increased to 1501.95 s, while the model reached a slightly improved maximum validation accuracy of 0.8124. The testing time decreased to 6.5 s, yet the model's performance on the test set was remarkably enhanced with a test accuracy of 0.9925 and perfect precision, recall, and *F*1 score, all at 0.9925. Throughout the 50 epochs, the training process demonstrated a consistent improvement in loss and accuracy metrics, particularly evident in the val_loss metric, which fluctuated but showed an overall improvement, reaching its lowest at Epoch 24 with a value of 0.67533. Several epochs exhibited model checkpoints where the val_loss improved and the model weights were saved due to the improved validation loss, indicating an effective learning process despite some fluctuations in val_accuracy.

### 5.7. ResNet152V2

**Figure 9:**
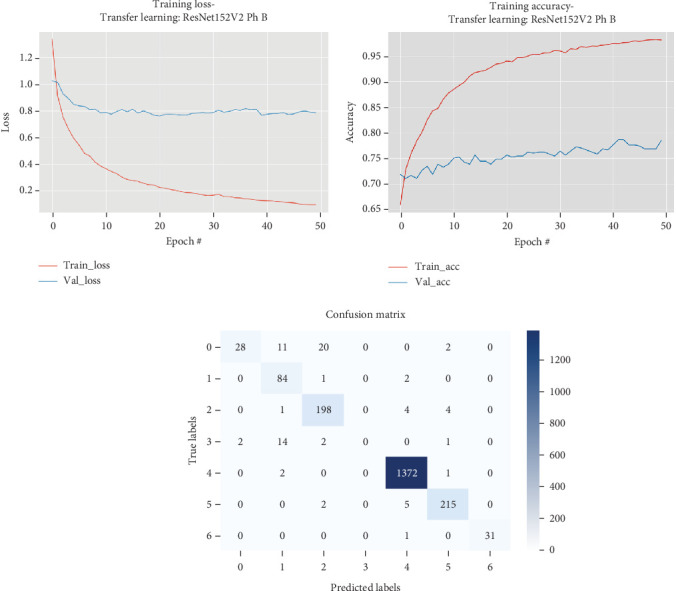
Accuracy and loss plots, along with the confusion matrix for the ResNet152V2 model.

The results for the ResNet152V2 model are presented in Table 8 and illustrated in Figure 9.

Table 8 and Figure 9 display that in Phase B of the fine-tuning process for the ResNet152V2 model, the model initially showed a gradual improvement in performance across the first several epochs. Starting with an initial validation loss of 1.02382 and a corresponding accuracy of 0.7186, the model's performance metrics steadily improved over the subsequent epochs. By the end of the training session, the validation loss had decreased to 0.76022 at Epoch 21, with a validation accuracy reaching up to 0.7665 by Epoch 36. The early improvement in loss and accuracy indicates that the model benefited from the fine-tuning process, which involved unfreezing all the layers and allowing the model to adjust its weights based on the training data. However, after Epoch 21, the validation loss fluctuated and did not significantly improve, suggesting that the model might have reached its optimal performance on the validation set under the current configuration.

### 5.8. MobileNet

**Figure 10:**
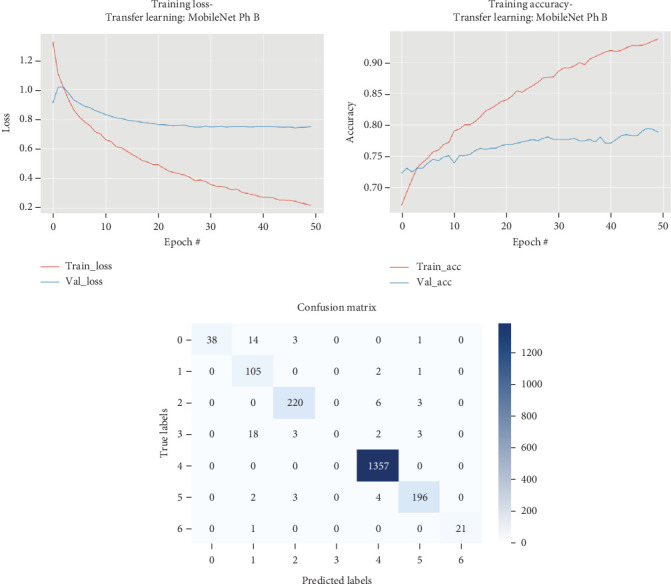
Accuracy and loss plots, along with the confusion matrix for the MobileNet model.

The results for the MobileNet model are presented in Table 9 and illustrated in Figure 10.

As evidenced by Table 9 and Figure 10, in Phase B of training the MobileNet model, all layers were fine-tuned, resulting in notable improvements in the model's performance over 36 epochs. Initially, the model's loss decreased significantly, demonstrating effective learning as the training progressed. The model's validation loss consistently improved, with the lowest val_loss recorded at 0.74755 by Epoch 34. The accuracy increased progressively, with a peak validation accuracy of around 0.7804 by the end of the 36th epoch.

The training began with a higher initial loss of 1.3275 and an accuracy of 0.6717. Through the epochs, as the model adjusted its weights and learned more complex features, both training and validation losses gradually decreased. Noteworthy is that the model's validation loss occasionally did not improve, as seen from epochs where “val_loss did not improve” was noted. However, the model generally showed a trend towards lower validation loss and higher accuracy, indicating effective learning.

Throughout the fine-tuning process, the model's accuracy steadily improved, reaching approximately 0.8992 by the 35th epoch, with the model performing optimally in terms of reducing loss and increasing accuracy. This phase of training highlights the effectiveness of fine-tuning all layers in MobileNet, significantly enhancing the model's capacity to learn and generalize from the data.

### 5.9. MobileNetV2

**Figure 11:**
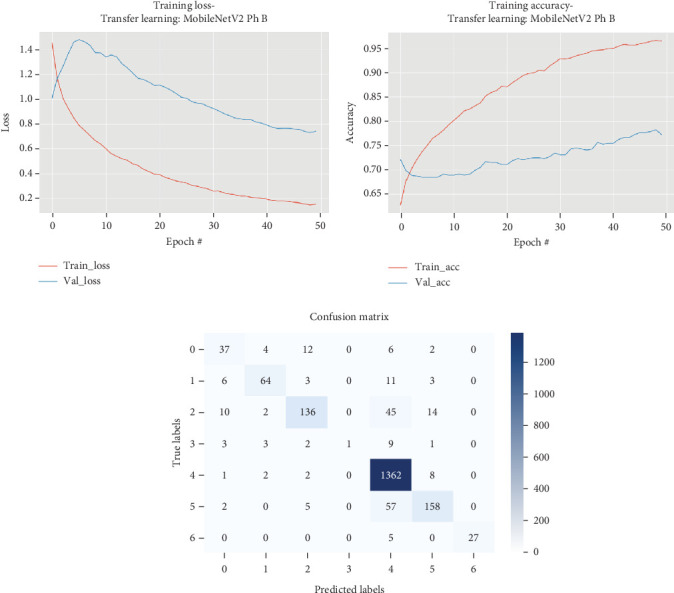
Accuracy and loss plots, along with the confusion matrix for the MobileNetV2 model.

The results for the MobileNetV2 model are presented in Table 10 and illustrated in Figure 11.

Table 10 and Figure 11 illustrate that the training process for the MobileNetV2 model, specifically during Phase B, where all layers were fine-tuned, involved extensive experimentation over 50 epochs. Initially, the model's performance was relatively modest, with improvements in both loss and accuracy occurring gradually. Early epochs showed fluctuating validation losses and slow gains in validation accuracy, indicating the complexity of fine-tuning a pretrained model. However, around the 27th epoch, significant progress was observed, as the model began consistently improving its validation loss, achieving a notable decline from 1.00675 to 0.83463. Concurrently, the validation accuracy showed a steady upward trend, ultimately reaching a competitive 74.05% by the 36th epoch.

The performance metrics from this phase reveal the impact of fine-tuning, with the final model delivering an accuracy of 93.98% on the training set and showing strong generalization with a validation accuracy of 74.05%. The training time for this phase was substantial, reflecting the intensive computations required for full-layer fine-tuning.

### 5.10. Test Score Comparison

**Figure 12:**
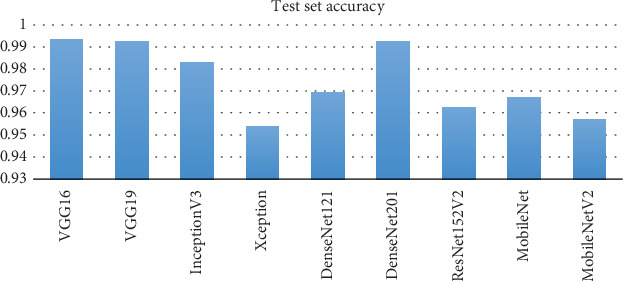
Test score comparison.

Figure 12 reveals that VGG16 and VGG19 achieve the highest accuracy, indicating their superior performance in the task. InceptionV3 and Xception, while still effective, show a notable decrease in accuracy compared to VGG models. DenseNet121 and DenseNet201 also perform well, with DenseNet201 slightly surpassing DenseNet121. ResNet152V2 and MobileNet offer competitive results, with ResNet152V2 performing slightly better. MobileNetV2, although effective, has the lowest accuracy among the models listed. Overall, VGG16 and VGG19 are the top performers, while other models like DenseNet and InceptionV3 provide strong alternatives, with the choice of model dependent on the trade-off between accuracy and computational efficiency.

### 5.11. Training Time Comparison

**Figure 13:**
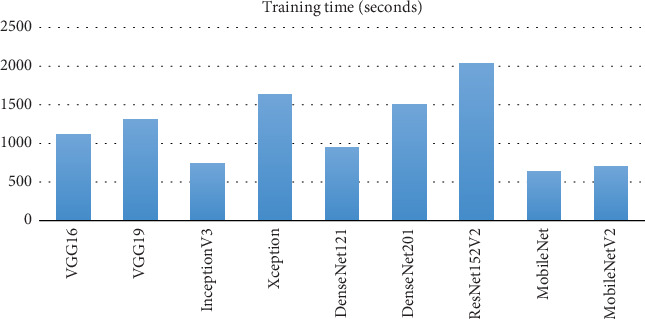
Training time comparison.

Figure 13 shows that the training time results for the skin cancer classification models highlight significant variability among them. MobileNet and MobileNetV2 have the shortest training times, taking 641.22 and 704.42 s, respectively, which underscores their efficiency in terms of training duration. InceptionV3 also demonstrates a relatively short training time of 735.8 s, reflecting its balanced complexity. VGG16, while achieving high accuracy, takes 1117.99 s for training, which is significantly longer than MobileNet and InceptionV3 but shorter than other high-performing models. VGG19 has a longer training time of 1309.95 s, yet it provides comparable accuracy to VGG16. DenseNet121 and DenseNet201 exhibit longer training times of 948.15 and 1501.95 s, respectively, with DenseNet201 taking notably more time. Xception and ResNet152V2 show the longest training times at 1636.99 and 2033.43 s, reflecting their complex architectures.

### 5.12. Overall Comparison

The results of the two-phase skin cancer classification reveal varied improvements across different models, with notable differences in performance metrics. VGG16 and VGG19 show substantial enhancements in test set accuracy in Phase B, reaching 0.993 and 0.992, respectively, compared to their Phase A scores of 0.857 and 0.853. This significant increase, accompanied by extended training times of 1117.99 s for VGG16 and 1309.95 s for VGG19, highlights the effectiveness of additional training. Similarly, DenseNet201 achieves an impressive test set accuracy of 0.992 in Phase B, up from 0.899 in Phase A, demonstrating a substantial improvement. InceptionV3 and Xception also show improved test set accuracy in Phase B, with InceptionV3 reaching 0.983 and Xception 0.954. Despite an increase in training time for Xception to 1636.99 s, the model shows enhanced precision and recall. MobileNet and MobileNetV2 exhibit increased test set accuracy in Phase B, with MobileNet reaching 0.967 and MobileNetV2 0.957, although the improvements are less pronounced compared to VGG and DenseNet models. Overall, VGG16 achieves the best test set accuracy of 0.993, underscoring the model's strong performance following extended training.

## 6. Conclusion

In conclusion, this paper presents a significant advancement in skin cancer classification through the development and application of a novel two-phase transfer learning model. By evaluating nine advanced pretrained models, our approach has demonstrated remarkable improvements in performance, as evidenced by superior accuracy and other critical metrics such as precision, recall, and *F*1 score. The comprehensive analysis reveals that our proposed model not only achieves a test set accuracy of 0.993 with VGG16 but also outperforms existing methods documented in the literature. This contribution represents a substantial step forward in the field of medical image analysis, offering a robust and effective solution for early skin cancer detection. Future work could focus on several key areas to build upon this research. First, expanding the dataset to include a wider variety of skin conditions and diverse demographic groups could enhance the model's generalizability and robustness. Second, integrating the model into a real-time diagnostic tool with user-friendly interfaces could facilitate its adoption in clinical settings. Finally, investigating the model's performance on other medical imaging tasks could provide insights into its versatility and broader applicability across different domains of healthcare.

## Figures and Tables

**Table 1:** Summary of previous studies on skin cancer detection models.

**Study**	**Model(s)**	**Best results**
Ayesha et al. [10]	MMF-SCD with VGG16, VGG19, and ResNet-50	Accuracy: 97.6%, precision: 97%, recall: 96%, and *F*1 score: 96%
Venugopal et al. [14]	Modified EfficientNetV2-M	Accuracy (binary): 95.95%, AUC: 0.98, and accuracy (multiclass): 94.80%
Alenezi, Armghan, and Polat [15]	WT-DRNNet with ResNet101 and ELM	Accuracy: 95.73%, specificity: 98.8%, precision: 95.84%, and *F*1 score: 93.44%
Jasil and Ulagamuthalvi [16]	Hybrid CNN with DenseNet and residual networks	Accuracy: 95%
Bozkurt [17]	Inception-ResNet-v2 with affine transformation data augmentation	Accuracy (augmented dataset): 95.09% and accuracy (original dataset): 83.59%
Tahir et al. [5]	DSCC_Net with CNN architecture and SMOTE Tomek technique	Accuracy: 94.17%, precision: 94.28%, recall: 93.76%, *F*1 score: 93.93%, and AUC: 99.43%
Aydin [18]	Histogram-based local descriptors with SVM and XGBoost	Accuracy: 96.50% and *F*1 score: 96.48%
Hosseinzadeh et al. [19]	Stacking approach with XGBoost, MLP, SVM, and random forest	Accuracy: 87.72% and sensitivity: 92.15%
Musthafa et al. [4]	Optimized CNN	Accuracy: 97.78%, precision: 97.9%, recall: 97.9%, and *F*1 score: 97.8%
Nagadevi, Suman, and Lakshmi [20]	AHC-EL with residual attention network, MobileNet, and Inception	Accuracy: 94.19% (HAM10000) and 97.33% (PH2)
Attallah [11]	Skin-CAD system, including faster R-CNN and ResNet	Accuracy: 98.80% (ISIC 2019), 97.2% (malignant vs. benign), and 85.4% (ISIC 2018)

**Table 2:** Performance metrics for the VGG16 model across the two phases.

**Phase**	**Training time (seconds)**	**Max validation accuracy**	**Testing time (seconds)**	**Test set accuracy**	**Precision**	**Recall**	**F**1** score**
A	436.7100	0.7844	2.6300	0.8572	0.8570	0.8572	0.8470
B	1117.9900	0.8503	1.9500	0.9935	0.9936	0.9935	0.9935

**Table 3:** Performance metrics for the VGG19 model across the two phases.

**Phase**	**Training time (seconds)**	**Max validation accuracy**	**Testing time (seconds)**	**Test set accuracy**	**Precision**	**Recall**	**F**1** score**
A	497.2400	0.7844	2.3000	0.8532	0.8574	0.8532	0.8436
B	1309.9500	0.8603	2.3200	0.9925	0.9925	0.9925	0.9925

**Table 4:** Performance metrics for the InceptionV3 model across the two phases.

**Phase**	**Training time (seconds)**	**Max validation accuracy**	**Testing time (seconds)**	**Test set accuracy**	**Precision**	**Recall**	**F**1** score**
A	250.0800	0.7485	3.7200	0.8308	0.8241	0.8308	0.8124
B	735.8000	0.7725	3.3100	0.9830	0.9831	0.9830	0.9830

**Table 5:** Performance metrics for the Xception model across the two phases.

**Phase**	**Training time (seconds)**	**Max validation accuracy**	**Testing time (seconds)**	**Test set accuracy**	**Precision**	**Recall**	**F**1** score**
A	410.9300	0.7565	2.8500	0.8537	0.8386	0.8537	0.8382
B	1636.9900	0.7545	2.6000	0.9538	0.9387	0.9538	0.9383

**Table 6:** Performance metrics for the DenseNet121 model across the two phases.

**Phase**	**Training time (seconds)**	**Max validation accuracy**	**Testing time (seconds)**	**Test set accuracy**	**Precision**	**Recall**	**F**1** score**
A	317.5600	0.7924	4.3400	0.8582	0.8576	0.8582	0.8470
B	948.1500	0.7864	3.8800	0.9695	0.9709	0.9695	0.9642

**Table 7:** Performance metrics for the DenseNet201 model across the two phases.

**Phase**	**Training time (seconds)**	**Max validation accuracy**	**Testing time (seconds)**	**Test set accuracy**	**Precision**	**Recall**	**F**1** score**
A	511.5900	0.8104	6.9700	0.8992	0.8983	0.8992	0.8944
B	1501.9500	0.8124	6.5000	0.9925	0.9925	0.9925	0.9925

**Table 8:** Performance metrics for the ResNet152V2 model across the two phases.

**Phase**	**Training time (seconds)**	**Max validation accuracy**	**Testing time (seconds)**	**Test set accuracy**	**Precision**	**Recall**	**F**1** score**
A	705.5800	0.7625	7.0400	0.8502	0.8504	0.8502	0.8288
B	2033.4300	0.7864	6.3100	0.9626	0.9561	0.9626	0.9561

**Table 9:** Performance metrics for the MobileNet model across the two phases.

**Phase**	**Training time (seconds)**	**Max validation accuracy**	**Testing time (seconds)**	**Test set accuracy**	**Precision**	**Recall**	**F**1** score**
A	144.95	0.8004	1.3200	0.8642	0.8473	0.8642	0.8482
B	641.22	0.7944	1.2300	0.9670	0.9581	0.9670	0.9609

**Table 10:** Performance metrics for the MobileNetV2 model across the two phases.

**Phase**	**Training time (seconds)**	**Max validation accuracy**	**Testing time (seconds)**	**Test set accuracy**	**Precision**	**Recall**	**F**1** score**
A	144.95	0.8004	1.3200	0.8642	0.8473	0.8642	0.8482
B	641.22	0.7944	1.2300	0.9670	0.9581	0.9670	0.9609

## Data Availability

The data that support the findings of this study are available at https://www.kaggle.com/datasets/kmader/skin-cancer-mnist-ham10000.
